# 1111. Therapeutic Drug Monitoring of Colistin in Cerebrospinal Fluid in the Treatment of Neurosurgical Meningitis caused by *Pseudomonas aeruginosa* and KPC-producing *Enterobacterales*

**DOI:** 10.1093/ofid/ofab466.1305

**Published:** 2021-12-04

**Authors:** Mohamad Yasmin, Amir nutman, Steven Marshall, Lu Wang, Ke Chen, Dafna Yahav, Jiping Wang, Jian Li, Robert A Bonomo

**Affiliations:** 1 Case Western Reserve University, Cleveland, Ohio; 2 Beilinson Hospital, Tel aviv, Tel Aviv, Israel; 3 Louis Stokes Cleveland Medical Center, Cleveland, OH; 4 Monash University, Melbourne, Victoria, Australia; 5 Tel Aviv University, Rabin Medical Center; 6 Louis Stokes Cleveland VA Medical Center, Cleveland, OH

## Abstract

**Background:**

Central nervous system (CNS) infections caused by carbapenem-resistant *Enterobacterales* (CRE) and Difficult-to-treat resistant (DTR)-*P. aeruginosa* (PA) present a therapeutic dilemma. Therapies are limited due to antibiotic resistance and inadequate CNS diffusion. Intraventricular polymyxins are utilized in this setting despite a lack in pharmacokinetic data after CNS injection. We describe the utilization of intravenous and intrathecal polymyxin E [colistimethate (CMS)] therapeutic drug monitoring (TDM) in 3 cases of post-neurosurgical meningitis.

**Methods:**

Bacterial identification and susceptibility testing were performed using MicroScan. TDM was employed by dosing CMS at 125,000 IU (i.e., 4.1 mg CBA or 10 mg) administered via external ventricular drain twice daily and 4.5 MIU (133.2 CBA or 360 mg) CMS administered over 30 minutes IV twice daily. Four pairs of CSF and blood samples were collected for each patient (Table 1). Samples were placed on ice to minimize in-vitro conversion of CMS to Colistin. Colistin binding in plasma and CSF was measured using ultracentrifugation. Concentrations of CMS and Colistin in CSF and human plasma were determined by liquid chromatography/mass spectrometry. Patients A, B and C received 20, 15, and 12 doses of CMS, respectively, prior to TDM.

**Results:**

Bacterial cultures revealed DTR *PA*, *bla*_KPC_*E. cloacae* and *bla*_OXA-48_*K. pneumoniae* for patients A, B and C, respectively. Colistin minimum inhibitory concentrations (MIC) were 0.5 µg/ml, 0.125 µg/ml, and 0.125 µg/ml, respectively. The measured CSF and plasma concentrations of CMS, Colistin, and binding are shown in Table 1. Clinical resolution and microbiological cure were attained in all patients.

Therapeutic Drug Monitoring of Unchanged CMS and Formed Colistin in CSF samples for patient A, B, and C

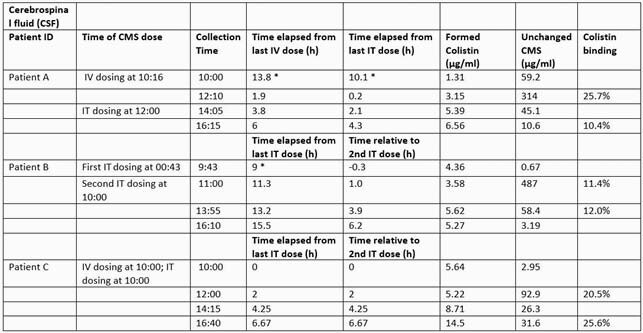

Therapeutic Drug Monitoring of Unchanged CMS and Formed Colistin in Plasma Samples for patient A, B, and C

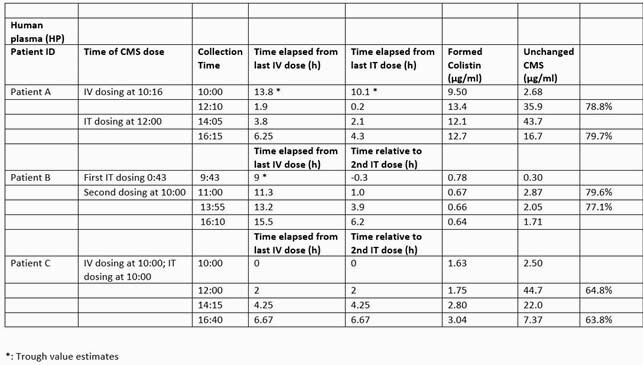

**Conclusion:**

Favorable concentrations of formed Colistin and CMS in CSF were achieved in 3 patients with complicated CNS infection. To the best of our knowledge, this is the first study to report the binding of Colistin in CSF in humans. A TDM method was effectively applied to demonstrate that Colistin achieves and maintains the PK/PD target (fAUC/MIC) [ratio of area under the plasma concentration curve of unbound drug to MIC] that best correlates with killing activity. Overall, our results support intraventricular polymyxins for treating DTR Gram-negative CNS infections.

**Disclosures:**

**Robert A. Bonomo, MD**, **entasis** (Research Grant or Support)**Merck** (Grant/Research Support)**NIH** (Grant/Research Support)**VA Merit Award** (Grant/Research Support)**VenatoRx** (Grant/Research Support)

